# OPTIMIZING GSA STUDENT CHAPTER NETWORKING ACROSS CAMPUS AND THE WORLD

**DOI:** 10.1093/geroni/igad104.0606

**Published:** 2023-12-21

**Authors:** Lauren Bouchard

**Affiliations:** Portland State University, Portland, Oregon, United States

## Abstract

The Portland State University (PSU) Gerontological Society of America Student Chapter was formed as a new student organization in 2022. Due to student interest and enthusiasm, this chapter was eager to invest in connections within PSU departments (e.g., public health, social work, and urban affairs) across Portland area campuses (e.g., Oregon Health and Science University) and between other student chapters internationally. Initially, this chapter focused on relationships between undergraduate and graduate students invested in aging research and education on PSU’s campus. Once a core group was formed, the chapter recruited members across campus, created an Aging 101 Service event to reach all students, and developed connections with professionals in the regional area. With guidance from the faculty advisor, this chapter reached out to the chapter at the University of Porto, the University of Aveiro in Porto, Portugal, to create a dual chapter networking event with colleagues in Portugal. This presentation will explain the chapter networking and provide suggestions to create regional, national, and international connections.

